# Accuracy of Conventional and Digital Radiography in Detecting External Root Resorption

**Published:** 2014-10-07

**Authors:** Abbas Mesgarani, Sina Haghanifar, Maryam Ehsani, Samereh Dokhte Yaghub, Ali Bijani

**Affiliations:** a *Department of Endodontics, Dental School, Babol University of Medical Sciences, Babol, Iran;*; b *Department of Oral and Maxillofacial Radiology, Dental School, Babol University of Medical Sciences, Babol, Iran;*; c *Dental Material Research Center, Dental School, Department of Endodontics, Babol University of Medical Sciences, Babol, Iran;*; d *Dental School, Babol University of Medical Sciences, Babol, Iran;*; e *Social Determinants of Health Research Center, Babol University of Medical Sciences, Babol, Iran*

**Keywords:** Dental Digital Radiography, Dental Radiography, Diagnostic Imaging, External Root Resorption, Root Resorption

## Abstract

**Introduction:** External root resorption (ERR) is associated with physiological and pathological dissolution of mineralized tissues by clastic cells and radiography is one of the most important methods in its diagnosis. The aim of this experimental study was to evaluate the accuracy of conventional intraoral radiography (CR) in comparison with digital radiographic techniques, *i.e.* charge-coupled device (CCD) and photo-stimulable phosphor (PSP) sensors, in detection of ERR. **Methods and Materials:** This study was performed on 80 extracted human mandibular premolars. After taking separate initial periapical radiographs with CR technique, CCD and PSP sensors, the artificial defects resembling ERR with variable sizes were created in apical half of the mesial, distal and buccal surfaces of the teeth. Ten teeth were used as control samples without any resorption. The radiographs were then repeated with 2 different exposure times and the images were observed by 3 observers. Data were analyzed using SPSS version 17 and chi-squared and Cohen’s Kappa tests with 95% confidence interval (CI=95%). **Result:** The CCD had the highest percentage of correct assessment compared to the CR and PSP sensors, although the difference was not significant (*P*=0.39). It was shown that the higher dosage of radiation increases the accuracy of diagnosis; however, it was only significant for CCD sensor (*P*=0.02). Also, the accuracy of diagnosis increased with the increase in the size of lesion (*P*=0.001). **Conclusion:** Statistically significant difference was not observed for accurate detection of ERR by conventional and digital radiographic techniques.

## Introduction

External root resorption (ERR) is a condition associated with physiological and pathological dissolution of mineralized tissues by odontoclastic cells [1, 2]. Early diagnosis is the key factor to detect and preserve the involved teeth [3]. Root resorption usually does not represent with any clinical sign or symptom. Hence, the diagnosis is generally based on its detection during radiographic examinations [2].

Numerous imaging modalities are currently accessible. Image acquisition is improved and is easier with the use of several tools that incorporate sensors using solid-state technology, *aka *charge-coupled device (CCD), or photo-stimulable phosphor (PSP) technology, which are known as a semi-direct or indirect acquisition modality [4-6]. However, the conventional intraoral film radiography (CR) is another option that compresses the three-dimensional anatomy into a two-dimensional image or shadowgraph, and thus greatly limits the diagnostic performance as the important features of the tooth and its surrounding tissues are detectable in the proximal plane (mesiodistal direction) only [7]. Similar features presenting in the buccolingual plane (*i.e.* the third dimension) may not be fully visible; however, this shortage could be overcome by taking several intraoral views at different angles [8].

CCD sensors and PSP plates are the intraoral digital radiographic techniques most commonly used in clinical dentistry for diagnosing different lesions [9]. Solid state detectors consist of a CCD or complementary metal oxide semiconductor (CMOS) chip that is sensitive to light and a scintillator layer that converts x-ray to light. The quality of the image produced by a solid state detector depends on dimensions of the chip pixel, type, and configuration of the scintillation layer, the electronics including analog-to-digital conversion, and the acquisition and display software. The CCD system uses a thin wafer of silicon as the basis for image recording [10], while PSP consists of a polyester base coated with crystalline halide emulsion composed of a europium-activated barium fluorohalide compound. PSP plates absorb and store x-ray energy, which is then released as phosphorescence upon stimulation by another light of an appropriate wavelength. Digital systems offer several advantages over conventional silver-halide analogue radiographic films, including reusability, reduced radiation dosage, being time-saving, possibility of image enhancement and ease of storage, retrieval and dentists’ communication [11]. Considering the importance of radiologic diagnosis of external root resorption and the potential difference in diagnostic performance of different imaging systems, the aim of this study was to evaluate the accuracy of CR with CCD and PSP sensors in detection of ERR in 3 different root surfaces including buccal, mesial and distal, with different cavity sizes and exposure times.

**Table1:** The number (N) and location of artificial root resorption in different groups (R: resorption detected, RN: resorption not detected)

**Group**	**Location**	**N**	**R (N)**	**RN (N)**
**1**	Buccal	10	10	20
**2**	Mesial	10	10	20
**3**	Distal	10	10	20
**4**	Buccal and mesial	10	20	10
**5**	Buccal and distal	10	20	10
**6**	Mesial and distal	10	20	10
**7**	Buccal, mesial and distal	10	30	0
**8**	No resorption (control group)	10	0	30

## Methods and Materials

In this experimental study, 80 extracted human mandibular premolars were collected. Teeth with root canal fillings, root resorption, fracture, cracks and incomplete apices, were excluded. The samples were divided into 8 groups (*n=*10).

After taking initial radiographies with conventional E-speed intraoral film (AGFA-Gevaert, Mortsel, Belgium), CCD sensor (DIXi3, planmeca Oy, Helsinki, Finland) and PSP sensor (Digora; soredex, Helsinki, Finland), the artificial defects similar to ERR were created using round diamond burs (Tizkavan, Tehran, Iran) with 0.8 mm, 1 mm, 1.2 mm and 1.4 mm diameters by drilling with the entire bur depth at apical half of the mesial, distal and buccal surfaces of the teeth and 10 teeth were placed in a control group without any resorption (Table1).

**Table 2:** Correct assessment of resorption (%) according to the surface of resorption and the radiological method, [confidence interval (CI)=95%] (CR=conventional radiography, CCD=charge-coupled device, PSP=photo-stimulable phosphor)

**Imaging method**	**Buccal **	**Mesial **	**Distal **	***P*** **-** **value**
**CR **	65.4	71	67.2	0.55
**CCD**	68.5	65	70	0.58
**PSP**	68.9	61.2	67.5	0.26

According to the factorial design rule, the number of usage of each bur was 30 times. All teeth were randomly numbered from 1 to 80 and the number, location and the size of cavities were listed and saved.

Teeth were separately repositioned in mandibular alveolar sockets of a cadaver skull that was borrowed with ethical approval from the Faculty of Dentistry, Babol University of Medical Sciences. The soft tissue was simulated by wax plates. Then the radiographs were repeated at 2 different exposure times; the exposure time for digital imaging (CCD and PSP) was 0.04 and 0.08 sec while this time for CR was considered 0.08 and 0.16 sec at 60 kVp. The CR films were processed in an automatic processor (HOPE dentamax, Warminster, PA, USA) based on manufacturers' recommendations. Digital intraoral images were taken using PSP sensors with 85-167 pixel size per µm (Digora; soredex, Helsinki, Finland) and a resolution of 6 LPM, and CCD sensor with 19 pixel size per µm (DIXi3, planmeca Oy, Helsinki, Finland) and 25 LPM resolution. The CR was also taken using intraoral E-speed size 2 films. The distance between the digital detectors or CR films and teeth were fixed by holders, the focus receptor distance was 30 cm.

The radiographic results were analyzed by three observers (a radiologist and two endodontists). The obtained films were evaluated using a light box and digital images were displayed on a 17-inch monitor (SyncMaster, Samsung, Seoul, Korea) without enhancement. The images were evaluated based on being able to determine the presence/absence and the surface of the defect. The reliability and degree of agreement was determined by means of Cohen's Kappa analysis with CI=95%. Correct detection and sensitivity (true positive) was defined as correct detection of a surface with defect, and specificity (true negative) was defined as correct detection of a surface without defect and positive and negative predictive value of the study was analyzed using the SPSS software (SPSS version 17.0, SPSS, Chicago, IL, USA) and the chi-square test.

## Results

A Total of 240 root surfaces were included in the study: 120 were detected with resorption and 120 were classified without any defect by the observers (Table 1). The number of cavities varied from 0-3 (0 in control group), and the location of the defects was considered in apical halves of buccal, mesial or distal surfaces. The cavity depths correlated to the bur size. The CCD had the highest rate of correct detection compared with the CR and PSP sensors, even though the difference was not significant (*P*=0.39).

**Figure 1:**
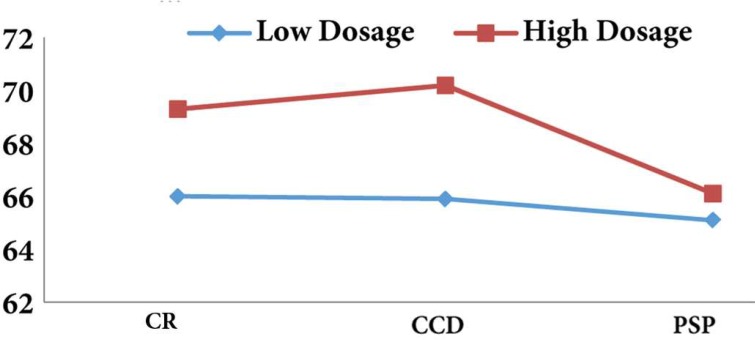
The percentage of correct assessment by the dosage and method of radiography (CR=conventional radiography, CCD=charge-coupled device, PSP=photo-stimulable phosphor)

Table 2 shows that the highest percentage of precise detection according to resorption surface and the radiological methods were observed in mesial, distal and buccal surfaces, in descending order, for the CR (*P*=0.55), CCD (*P*=0.58) and PSP (*P*=0.26) sensors, respectively.

**Figure 2:**
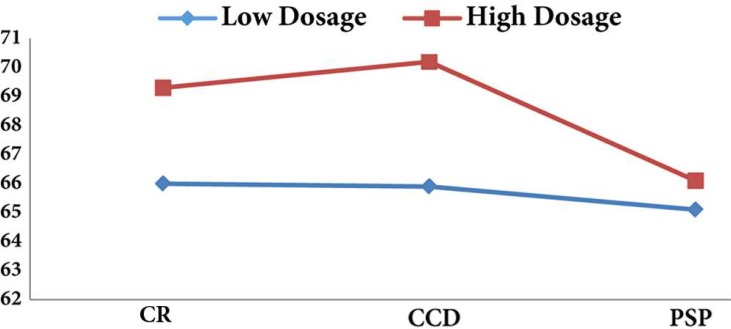
The percentage of precise assessment according to the cavity size (CR=conventional radiography, CCD=charge-coupled device, PSP=photo-stimulable phosphor)

According to the results, high dosage of radiography increases the accuracy of diagnosis (Figure 1); however, this issue is only significant for CCD sensors (*P*=0.02). Figure 2 also, shows that the surface without cavity has the highest accuracy of diagnosis, also the accuracy of diagnosis increases with the increase in cavity size (*P*=0.001). The results revealed that the most sensitivity and specificity for high exposure time of CCD sensor were 81.4 and 68.2 while for lower amount of exposure time they were 78.7 and 66.9, respectively. In addition, the highest kappa coefficient was for high exposure time (0.458±0.055).

Moreover, regarding PSP sensor, the highest sensitivity and specificity of high exposure time were 79.7 and 63.9 while they were 82.4 and 66.2 for the low exposure time.

Also, the highest kappa coefficient was related to high exposure time and this was 0.458±0.055.

## Discussion

This experimental study proved that there is no significant differences in detection of ERR with variable sizes and in different tooth surfaces between conventional and digital intraoral radiographic techniques. 

The diagnosis of ERR it highly important as it can increase the chance of treatment and maintenance of the tooth [12]. According to the impediments of CR, recently the digital radiographic techniques such as CCD and PSP have gained notable acceptance among the clinicians. 

Although the CCD technique showed the highest amount of efficiency, but the difference in the accuracy of assessments between the conventional and digital radiographic methods was not significant. In the study by Kamburoğlu *et al. *[10], CCD and CR revealed more correct readings than PSP. It seems that the low accuracy of PSP is due to the quality of the phosphor plate, low resolutions, and low signal-to-noise ratio and the mechanism of the scan. Borg *et al.* [13] showed that digital radiography has similar sensitivity to CR in resorption diagnosis but the amount of radiation is lower in digital radiography. Nevertheless, digital radiography has some advantages that CR does not, for instance the images can be manipulated such as enlargement, inversion, and contrast enhancement [14]. Contrary to this study, Westphalen *et al.* [15] have shown that the sensitivity of digital radiographic method was statistically higher than the CR.

Similar to the findings of Levander *et al.* [3] and Borg *et al.* [13], the percentage of correct assessment is increased by the size of the cavities in this study. Removing the larger amount of dental tissue leads to a wider radiolucent area. Therefore, the root resorption diagnosis rate is higher for larger cavities by both conventional and digital radiographic methods.

In contrast to the present study, Shokri *et al.* [2] showed no significant differences in detection of resorptive cavities with different sizes among cone-beam computed tomography (CBCT), CCD and CR methods.

In this study there was not any significant differences between detection of the ERR in buccal, mesial and distal surfaces of the root. In another study, the accurate assessment was related to the proximal surfaces with no difference in the diagnosis of the cavities in cervical, middle and apical portion of the root [16]. Kamburoğlu *et al.* [10] showed that the most difficult surface of the root for resorption diagnosis are the buccal and proximal aspects in apical areas while the proximal, cervical and the medium surfaces had the most accurate readings. According to the study by Shokri *et al.* [2], CBCT did not show any significant supremacy in cavity detection, compared to other methods except for cavities in the apical area.

**Table 3:** The sensitivity, specificity and Kappa coefficient of each observer by the time of exposure for three radiological methods (CR=conventional radiography, CCD=charge-coupled device, PSP=photo-stimulable phosphor, ET=exposure time, NPV=negative predictive value, PPV=positive predictive value)

**Method**	**ET (sec)**	**Observer**	**Sensitivity (%)**	**Specificity (%)**	**NPV (%)**	**PPV (%)**	**Kappa (SE)**	***P*** **-** **value**
**CR**	0.16	1	82.6	70.3	86.7	63.3	0.5 (0.052)	0.001
		2	65.8	63.6	68.3	60.8	0.292 (0.062)	0.001
		3	73.9	64.9	80	56.7	0.367 (0.058)	0.001
	0.08	1	77.9	61	87.5	44.2	0.317 (0.055)	0.001
		2	80	53.3	86	65	0.4 (0.056)	0.001
		3	73.2	43.3	84.2	59.8	0.275 (0.057)	0.001
**CCD **	0.08	1	77.7	60.7	87.7	43.3	0.308 (0.055)	0.001
		2	81.4	67.5	86.7	58.3	0.45 (0.055)	0.001
		3	80.9	68.2	85.8	60	0.458 (0.055)	0.001
	0.04	1	66.7	57.1	80	40	0.2 (0.058)	0.001
		2	71.7	63.5	78.3	55	0.323 (0.059)	0.001
		3	78.7	66.9	84.2	58.3	0.425 (0.057)	0.001
**PSP**	0.08	1	79.7	59.7	90	39.3	0.292 (0.055)	0.001
		2	76.6	62.6	85	49.2	0.342 (0.057)	0.001
		3	63.6	63.9	63.3	64.2	0.458 (0.055)	0.001
	0.04	1	82.4	58.7	92.5	35	0.275 (0.062)	0.001
		2	76.4	61.5	85.8	45.8	0.317 (0.056)	0.001
		3	77.7	66.2	83.3	57.5	0.408 (0.057)	0.001

Table 3 shows that the most sensitivity and specificity rates of CR for the higher exposure times are 82.6 and 70, respectively and for the lower exposure time are 80 and 61, respectively. The most Kappa coefficient value was dedicated to the higher exposure time (0.5±0.052). Similar to the results of Borg *et al.*’s study [13], this investigation has shown a higher percentage of correct reading for all of the radiographic methods using higher exposure times.

In some studies it was found that the angulation of radiography has an important role in correct detection of the resorption. In the study by Westphalen *et al.* [15], radiographic images of teeth were taken in orthoradial, mesial and distal angulations and it was found that for the cavities which were not visible in orthoradial images, changing the horizontal angles can increase the chance of their detection. Also, some cavities were detectable by images taken with mesial and distal angulation which was similar to the results revealed by Borg *et al.* [13] and Andreasen *et al. *[16]. In comparison to these studies Kamburoğlu *et al.* [10] obtained a higher correct detection rate by the orthoradial angulations rather than distoradial and mesioradial. However, the most correct detection was achieved when the images from all angulations were evaluated simultaneously.

## Conclusion

There was no significant difference between conventional and digital radiographic methods in terms of detecting external root resorption.
